# A Successful Porro-Cesarean Section

**Published:** 1890-08

**Authors:** 


A Successful Porro-Cesarean Section. — Dr. Joseph Price, of Philadelphia, has recently made a successful Porro operation, the history of which possesses peculiar interest. A healthy woman, forty-four years old, had a pelvic fibroid, 6 x 4 inches in diameter, completely blocking the parturient canal, the uterus being studded with numerous smaller fibroids, from the size of a filbert to an egg. The operator waited for term and chose, as nearly as could be estimated, the last day. After incising the uterus and delivering the child, a large healthy female, the uterus and tumor were lifted out, a Bantock-Koeberle noeud and pins were applied, and all cut away. The intestines were kept from view by large flat sponges, and the sutures placed without allowing a drop of fluid to enter the abdomen. On the ninth day, the stitches having been removed, the patient was nursing her baby, bright and cheerful, having had no discomfort. This was a perfect supra-vaginal amputation, with extraperitoneal treatment of the pedicle, and as near an ideal operation as surgery can boast.

Dr. Price has now done a series of twenty-five consecutive supra-vaginal hysterectomies, including this Porro, without a death — the most perfect series, so far as we know, yet reported.

---

The first fruits of the new medical law are beginning to appear. The annual announcements of Bellevue Hospital Medical College, and the Medical Department of the University of the City of New York, just received, state that "with the session of 1891-'92 three courses of lectures will be required for graduates." We expect that other colleges not heretofore requiring three courses will fall into line as gracefullyas do these two metropolitan schools.

